# Concise Review: Kidney Stem/Progenitor Cells: Differentiate, Sort Out, or Reprogram?

**DOI:** 10.1002/stem.486

**Published:** 2010-07-22

**Authors:** Oren Pleniceanu, Orit Harari-Steinberg, Benjamin Dekel

**Affiliations:** a Pediatric Stem Cell Research Institute, Sheba Medical CenterTel Hashomer, Israel; b Sheba Center for Regenerative Medicine, Sheba Medical CenterTel Hashomer, Israel; c Sackler Faculty of Medicine, Tel Aviv UniversityTel Aviv, Israel; d Departments of Pediatrics, Sheba Medical CenterTel Hashomer, Israel; e Pediatric Nephrology, Sheba Medical CenterTel Hashomer, Israel

**Keywords:** Adult stem cells, Kidney, Cell surface markers, Cellular therapy, Developmental biology, Embryonic stem cells, Fetal stem cells

## Abstract

End-stage renal disease (ESRD) is defined as the inability of the kidneys to remove waste products and excess fluid from the blood. ESRD progresses from earlier stages of chronic kidney disease (CKD) and occurs when the glomerular filtration rate (GFR) is below 15 ml/minute/1.73 m^2^. CKD and ESRD are dramatically rising due to increasing aging population, population demographics, and the growing rate of diabetes and hypertension. Identification of multipotential stem/progenitor populations in mammalian tissues is important for therapeutic applications and for understanding developmental processes and tissue homeostasis. Progenitor populations are ideal targets for gene therapy, cell transplantation, and tissue engineering. The demand for kidney progenitors is increasing due to severe shortage of donor organs. Because dialysis and transplantation are currently the only successful therapies for ESRD, cell therapy offers an alternative approach for kidney diseases. However, this approach may be relevant only in earlier stages of CKD, when kidney function and histology are still preserved, allowing for the integration of cells and/or for their paracrine effects, but not when small and fibrotic end-stage kidneys develop. Although blood- and bone marrow-derived stem cells hold a therapeutic promise, they are devoid of nephrogenic potential, emphasizing the need to seek kidney stem cells beyond known extrarenal sources. Moreover, controversies regarding the existence of a true adult kidney stem cell highlight the importance of studying cell-based therapies using pluripotent cells, progenitor cells from fetal kidney, or dedifferentiated/reprogrammed adult kidney cells. Stem Cells 2010; 28:1649–1660.

## THE CLINICAL PROBLEM

The prevalence of chronic kidney disease (CKD) and end-stage renal disease (ESRD) is dramatically increasing [1], and at the same time, the Medicare cost of ESRD has risen from $12.2 in 2000 to $20.8 billion in 2007 [2]. ESRD is incurable, requiring renal replacement therapy, that is, dialysis or preferably renal transplantation. However, the shortage of available organs for transplantation continues to severely limit this option [3].

How can organ shortage be combated? In general, supply of organs can be increased, or their demand can be decreased.

When considering cell replacement in diseased kidneys via cell transfer, one should carefully dissect the timing of such a therapy, as end-stage kidneys are already small and fibrotic and would therefore not allow for the incorporation of cells or for their paracrine effects. Thus, late CKD stages warrant whole kidney replacement, independent of the native kidneys, leading to a need for increased organ supply. Accordingly, we have previously demonstrated that stage-specific human and porcine embryonic kidney tissue can remarkably grow, differentiate, and undergo vascularization, achieving successful organogenesis of urine-producing miniature kidneys in immuno-deficient animals [4–6]. The “growing kidneys” concept is suggested to be applicable to ESRD as whole kidney replacement, affording an additional source of kidney tissue [5]. Other approaches include porcine organ xenografts [7] or bioengineering of histocompatible renal units [8]. In the case of whole kidney replacement by generating kidneys de novo, the generated organ will have to produce sufficient glomerular filtration rate (GFR) to support body homeostasis. As this is a difficult task, it will likely be easier to approach organ shortage by decreasing organ demand.

Unlike ESRD, earlier stages of CKD, when residual function and histology are partially preserved, are expected to be more suitable for cell therapy, aiming at halting progression of CKD to ESRD. In this scenario, progressive kidney damage/fibrosis may lead to demands on healthy segments, creating a pathway of unrelenting damage over time. However, the tempo of decline may be decreased by serial interventions. Stem cells, able to self-renew and to intervene in building/maintaining the structural and functional integrity of tissues, are especially attractive for such a purpose.

Because CKD is composed of multiple etiologies in which different kidney cell types are affected (glomerular and tubular epithelium, glomerular and peri-tubular capillaries, interstitial cells), defining the effect of specific stem cells on a particular mature cell type can link various modes of cell therapy to diverse clinical applications. For instance, podocyte loss in many glomerular diseases, such as focal segmental glomerulosclerosis, persistent peri-tubular endothelial injury and dysfunction in the hemolytic uremic syndrome, and proteinuric states (for which proximal tubular cells are especially susceptible), are likely to benefit from different types of stem/progenitor cells. However, this categorization may become irrelevant once all etiologies succumb to the common pathological final pathway of progressive renal injury [9].

## DEVELOPMENTAL NEPHROLOGY AS A BASIS FOR THERAPEUTIC APPLICATIONS

**Figure 1:**
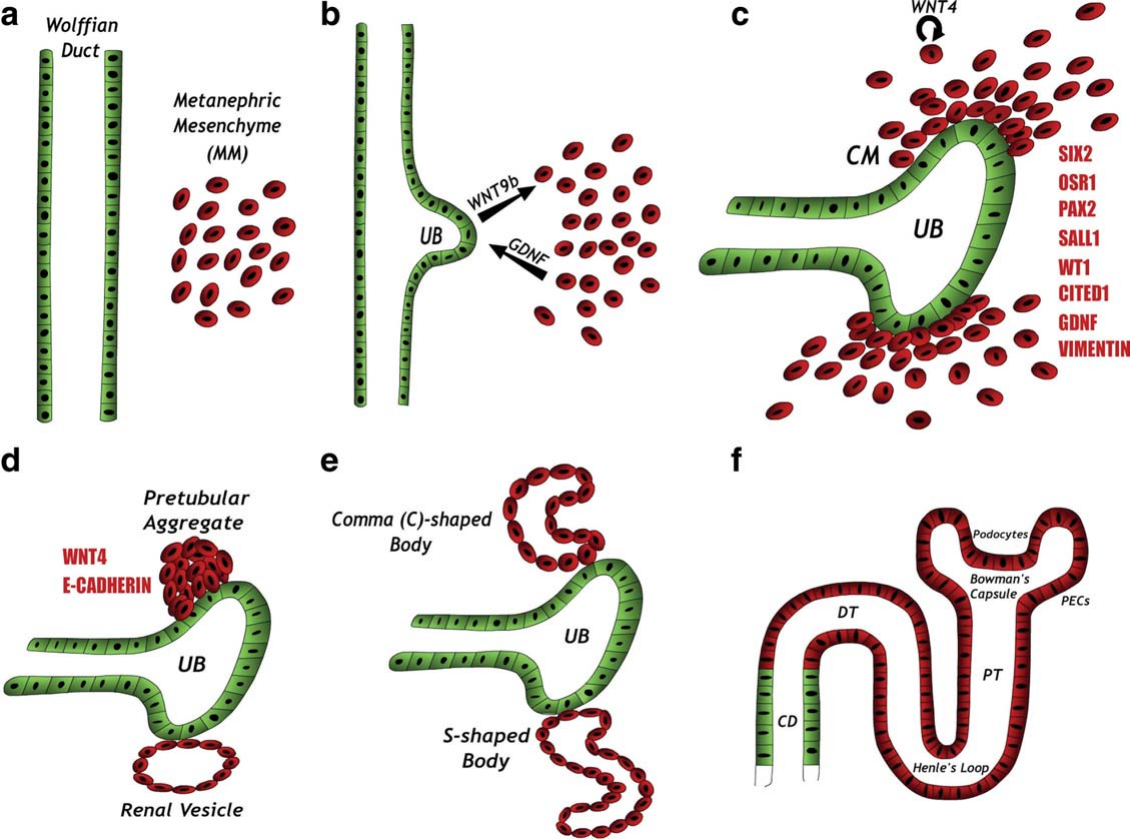
Kidney development. **(A):** The kidney is formed via reciprocal interactions between two precursor tissues derived form the intermediate mesoderm: the Wolffian duct and the MM. **(B):** MM-derived signals, mainly the glial-derived neurotrphic factor, induce an outgrowth from the Wolffian duct, termed the UB. The UB then invades the MM and secretes WNT9b, thereby attracting MM cells. **(C):** MM cells condense around the tips of the branching UB, forming the condensed or CM. The CM expresses a unique combination of genes (red) and the mesenchymal marker, vimentin. The CM contains the kidney stem cells and is capable of self-renewal. In response to UB signals, CM cells start to produce WNT4, which acts in an autocrine fashion, leading to epithelialization of the cells. **(D–F):** The induced cells acquire an epithelial phenotype. This change is accompanied by the shutting down of the major transcription factors described before **(B)** and by the acquisition of the epithelial marker E-cadherin. The cells sequentially form the pretubular aggregate, renal vesicle, C-, and S-shaped bodies, and finally the mature nephron. The cells derived from the CM form most of the nephron body (from glomerulus to distal tubule), whereas the UB-derived cells form the collecting duct. Abbreviations: CD, collecting duct; CM, cap mesenchyme; DT, distal tubule; PECs, parietal epithelial cells; PT, proximal tubule; UB, ureteric bud.

**Figure 2:**
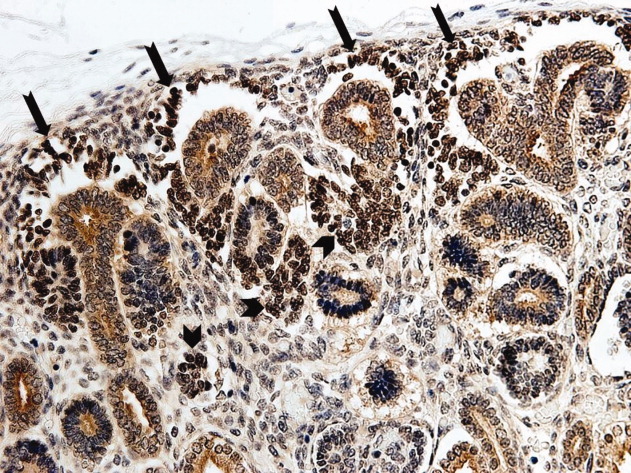
SIX2 immunostaining in human fetal kidney: SIX2, playing a major role in the self-renewal of the nephron's stem/progenitor cells, is seen here localizing to the MM, predominantly to the cap mesenchyme (arrows), and also to some tubular derivatives (arrowheads). This corresponds to the findings in mice [15], where it was shown that by 15.5 days postcoitum, SIX2 expression is restricted to the cap mesenchyme and early pretubular aggregates. SIX2 expression ceases 34 weeks postgestation in humans and in the immediate postnatal period in mice, leading to exhaustion of the stem cell pool and lack of true regenerative capacity (The figure obtained from [17]).

**Figure 3:**
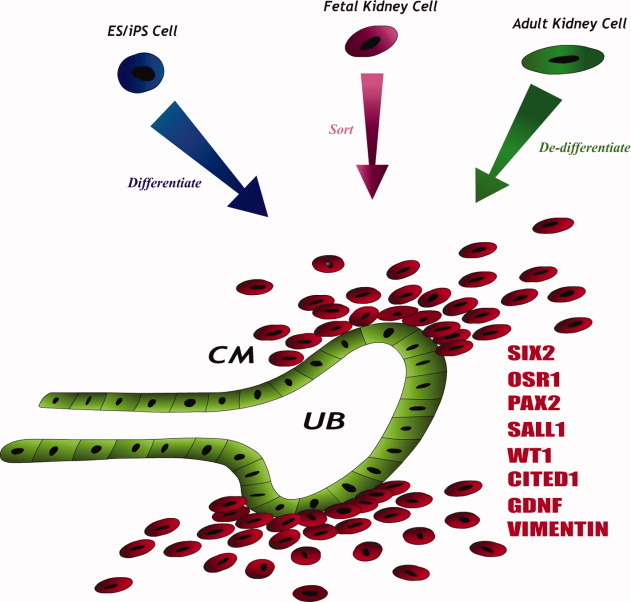
Regenerating nephrons: The cap mesenchyme cells (red) are the main players toward the ultimate goal of renal regenerative medicine and therefore different strategies are envisioned to obtain these cells or create an equivalent population of cells with nephrogenic potential: differentiation from pluripotent cells (ESCs or iPS cells), sorting of these cells from human fetal kidneys and de-differentiation via genetic reprogramming of adult kidney cells. Abbreviations: CM, cap mesenchyme; ESCs, embryonic stem cells; iPS, induced pluripotent stem cell; UB, ureteric bud.

The metanephros, the mature mammalian kidney, is formed via reciprocally inductive interactions between two precursor tissues, which are derived from the intermediate mesoderm (IM), that is, the metanephric mesenchyme (MM) and the ureteric bud (UB), a derivative of the Wolffian duct [10,11]. This complex process is summarized in Figure 1. MM cells that condensate and maintain themselves at the tips of the UB, giving off cells that differentiate into nephrons [12], are especially important. Recent experiments [13–16] have established that these progenitor cells in the condensed or cap mesenchyme (CM) fulfill the criteria of a true committed stem cell, capable of self-renewing and differentiating into different types of nephron epithelia. Prior to UB induction, the CM expresses a unique combination of transcription factors, including the *Hox11* paralogs, *Osr1*, *Pax2*, *Eya1*, *Wt1*, *Six2*, *Sall1*, and *Cited1* [10], considered early markers of kidney progenitor cells (Fig. 1). Among these markers, it was shown that continued expression of *Six2* is required for self-renewal of this stem cell population as nephrogenesis continues (Fig. 2) [15]. Interestingly, *Osr1* has been recently shown to mark an even earlier lineage in the IM, capable of giving rise to all metanephric cell components, including the Six2+ epithelial nephron progenitors, renal vasculature, and smooth muscle cells [16]. Notably, silencing of most of these genes coincides with termination of nephrogenesis (human, 34th gestational week; mice, 2 weeks postnatal) [18,19]. As a result, endowment of new nephrons is restricted to prenatal development in humans, and to the first 2 weeks after birth in rodents [20]. Therefore, the ultimate goal of renal regenerative medicine is to isolate and/or create an unlimited supply of human cells resembling the renal progenitors residing in the MM or CM, harboring true nephrogenic potential, to regenerate and replenish epithelial cell types within the nephron. Theoretically, the nephron stem/progenitor pool can be differentiated from pluripotent cells, sorted out from the developing kidney, reverted or dedifferentiated from adult kidney cells, or transdifferentiated from nonrenal cells (Fig. 3). However, in light of the difficulties in locating such cells, especially in humans, utilizing nonspecific extrarenal stem cells should be considered. For example, hematopoietic stem cells (HSCs), endothelial progenitor cells (EPCs)/hemangioblasts, and multipotent mesenchymal stromal cells (MSCs), are stem cells completely devoid of nephrogenic potential [21–24], but may enhance the intrinsic reparative capabilities of the kidney. As EPCs/hemangioblasts have been shown to possess vasculogenic/angiogenic potential in various organs, and specifically in the kidney [21–26], they can potentially restore the damaged microvasculature and reverse tissue hypoxia. The latter are two crucial factors in the chain of events leading to kidney fibrosis and CKD, and if restored by cell therapy may in turn heal nephron epithelia [27].

**Figure 4:**
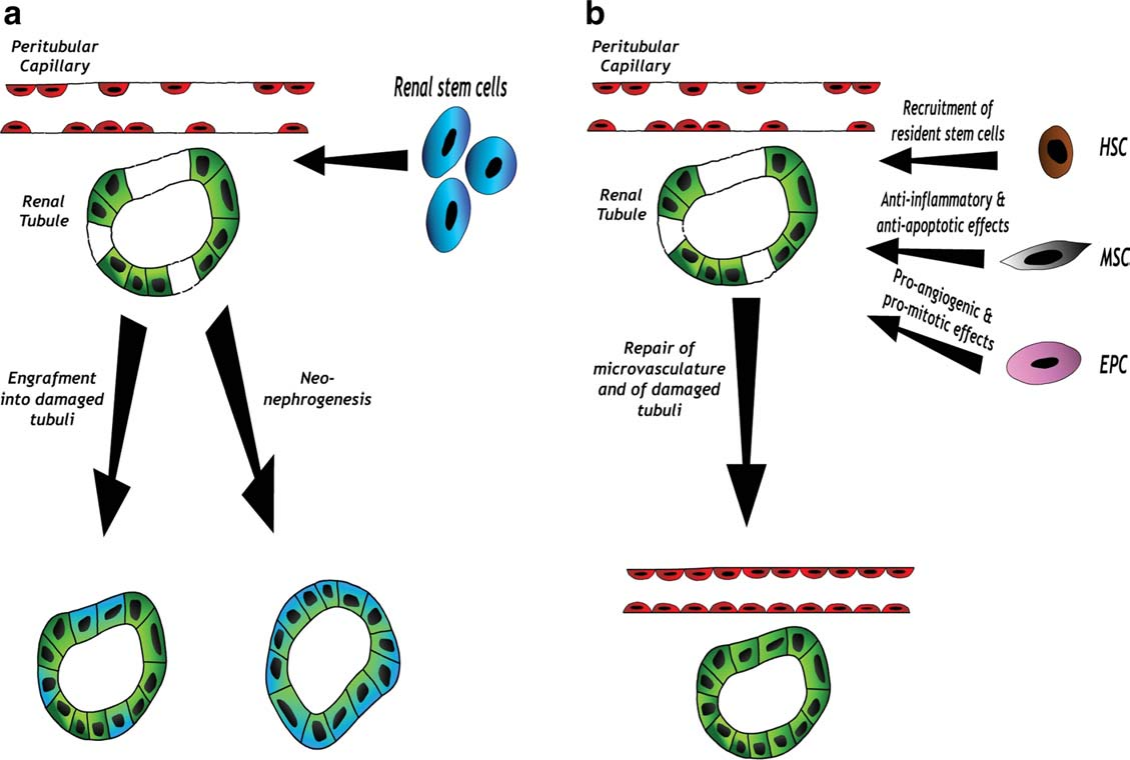
Two strategies for kidney repair after injury: **(A):** Truly committed renal stem cells (blue) harbor nephrogenic potential and contribute to kidney regeneration via engraftment into damaged tubuli and differentiation into tubular cells (left: blue-green tubular cells that originated from stem cells) and also by the creation of new nephrons (neo-nephrogenesis; right: cells in the new tubule originate from the stem cells and are therefore all blue-green). **(B):** Various extrarenal stem cells (HSCs, MSCs, EPCs) can assist kidney repair through different paracrine effects, possibly leading to the restoration of the damaged microvasculature, thereby allowing the surviving tubular cells to proliferate and reconstitute a functioning tubule (all cells in the repaired tubule are green, originating from the surviving cells). Abbreviations: EPC, endothelial progenitor cell; HSC, hematopoietic stem cell; MSC, mesenchymal stromal cell.

Therefore, both renal and nonrenal stem cells can be utilized for kidney repair potentially operating via differentiation-dependent (Fig. 4A) and differentiation-independent mechanisms (Fig. 4B), respectively. Although we hypothesize that a combination of the two approaches might be the optimal way of using stem cells for kidney regeneration, this review focuses on the first approach, summarizing the options for obtaining genuine renal stem/progenitor cells.

## DIFFERENTIATION OF RENAL PROGENITORS FROM PLURIPOTENT STEM CELLS

Pluripotent mouse and human embryonic stem cells (ESCs) and induced pluripotent stem (iPS) cells can theoretically give rise to all cell types in the body, and therefore carry renal potential. In fact, both undifferentiated and differentiated renal tissue has been observed in teratomas induced in immunodeficient mice after mouse and human ESCs injection [28,29]. However, there are several major limitations to the use of ESCs for kidney regeneration. The first issue regards the ethical, political, and religious problems surrounding the use of cells derived from early embryos. In addition, a major concern is the mal-differentiation of the cells into unwanted tissues or even the formation of teratomas (see, e.g., Supporting Information Fig. 1).

To avoid this danger, one must direct the cells to a state of differentiation that will on the one hand provide them with the potential to regenerate mature kidney cells of various types and on the other hand prevent mal-differentiation. This can be achieved by the controlled activation of the correct network of nephric transcription factors (see above, Developmental nephrology). Unfortunately, attaining this exact state of differentiation in vitro has proven to be quite difficult. Many attempts have been made to induce pluripotent cells in this manner, applying both growth factor combinations [bone morphogenetic protein (BMP)/Activin/Retinoic acid] and genetic approaches [30–36]. However, most differentiation studies, even after successfully inducing renal lineage genes, failed to pinpoint the exact stage in nephrogenesis (IM, MM, CM) to which ESCs were differentiated along the renal lineage. In addition, analysis of the induced cells in functional in vivo models is lacking from most reports, in sharp contrast to human ESCs-derived central nervous system or cardiac progenitors tested in relevant disease models [37,38]. An exception is described in a report on beneficial effects of murine ESCs in a genetic mouse model of Alport syndrome [39], where undifferentiated ESCs injected into Col4α3-deficient mice significantly improved renal function and histology. However, because the authors used undifferentiated ESCs cells rather than ESCs-derived renal progenitors, their findings are limited to translation.

Defining human ESC-derived renal progenitors, assessment of their repopulation ability and their in vivo function is especially important as this could ultimately pave the way to utilization of human iPS cells as an unlimited source of cells for renal regeneration or modeling of human disease in which renal progenitors are perturbed (e.g., renal dysplasia). A major advantage of ESCs-derived renal progenitors is their being autologous rather than allogeneic [40], a fact that would circumvent many of the ethical issues surrounding the use of ESCs.

## ISOLATION OF RENAL PROGENITORS FROM DEVELOPING KIDNEYS

As nephrogenesis progresses, the relative proportion of the nephrogenic zone decreases. However, due to the fact that stem cells are present in the embryonic kidney until relatively late in gestation, can be exploited for their isolation, making the fetal kidney an attractive source for isolation and utilization of tissue-specific stem cells [17,41]. Three main approaches have emerged for this purpose: (a) Transplantation of whole embryonic kidneys or fetal tissue including of human origin (discussed elsewhere [4–6,41]). (b) Transplantation of heterogeneous populations of fetal cells. (c) Transplantation of specific renal embryonic stem/progenitor cell populations.

### Heterogeneous Fetal Kidney Cells

Encouraging results regarding the use of cells from developing kidneys came from a report demonstrating that transplantation of a heterogeneous population of dissociated E14.5 and E17.5 rat fetal kidney cells under the kidney capsule lead to the creation of renal structures, and had beneficial effects on kidney function in a 5/6 nephrectomy model of kidney injury [42]. The same group also showed [43] that similarly to whole organ transplants [4], the gestational age of cells to be transplanted has to be chosen carefully, as early fetal kidney (E14.5) cells differentiated to nonrenal tissues, whereas cells from later gestational stages showed poor ability to form kidney structures. Kim et al. [44] recently showed that E17.5 rat fetal kidney cells were able to reconstitute kidney tissues only when cultured through passage one, whereas P2 cells experienced proliferation arrest and apoptosis, leading to poor regenerative potential in vivo. This finding underscores the importance of defining culture conditions that will minimize cellular stress and enable cell expansion to obtain clinically relevant amount of cells.

### Isolated Populations of Embryonic Kidney Progenitors

Few attempts have been made to characterize and use specific progenitor/stem populations from the developing kidney.

Lazzeri et al. [45] relied on the previously reported adult parietal epithelial multipotent progenitors (APEMP), characterized by the expression of CD24 and CD133 [46]. Based on the assumption that these putative adult progenitors are a remnant of a similar embryonic population, the same CD24+CD133+ phenotype was used to sort out cells from human embryonic kidney. It was demonstrated that this population initially localizes to the MM, representing 35%–50% of kidney cells, gradually decreasing in size and becoming restricted to the urinary pole of the Bowman capsule, possibly persisting into adulthood as the APEMPs. However, lineage tracing was not performed and therefore the association between the adult and embryonic populations has not yet been validated. Importantly, these cells incorporated into tubules of SCID mice with glycerol-induced acute renal failure and differentiated into various types of renal cells.

### Identification of Renal Stem/Progenitor Markers for Cell Selection Strategies

The fact that specific surface markers on stem/progenitor cells in the kidney have not yet been identified hampers the identification of these cells in the developing kidney [47].

One approach to identify surface markers is through the use of global gene expression analysis, which in the case of murine embryonic kidney has identified *CD24a* (different from the human *CD24*) and *Cadherin11* as MM surface markers [48].

To look for such markers in the human kidney, we analyzed the developing human kidney concomitant with the pediatric renal malignancy Wilms' tumors (WT) using microarrays [49]. WT results from differentiation arrest of embryonic progenitor cells committed to the nephric lineage accumulating in the tumor as undifferentiated blastema. Nevertheless, because the differentiation arrest is only partial, differentiated epithelial (tubular-like) and stromal elements are also observed in the tumor [50].

**Figure 5:**
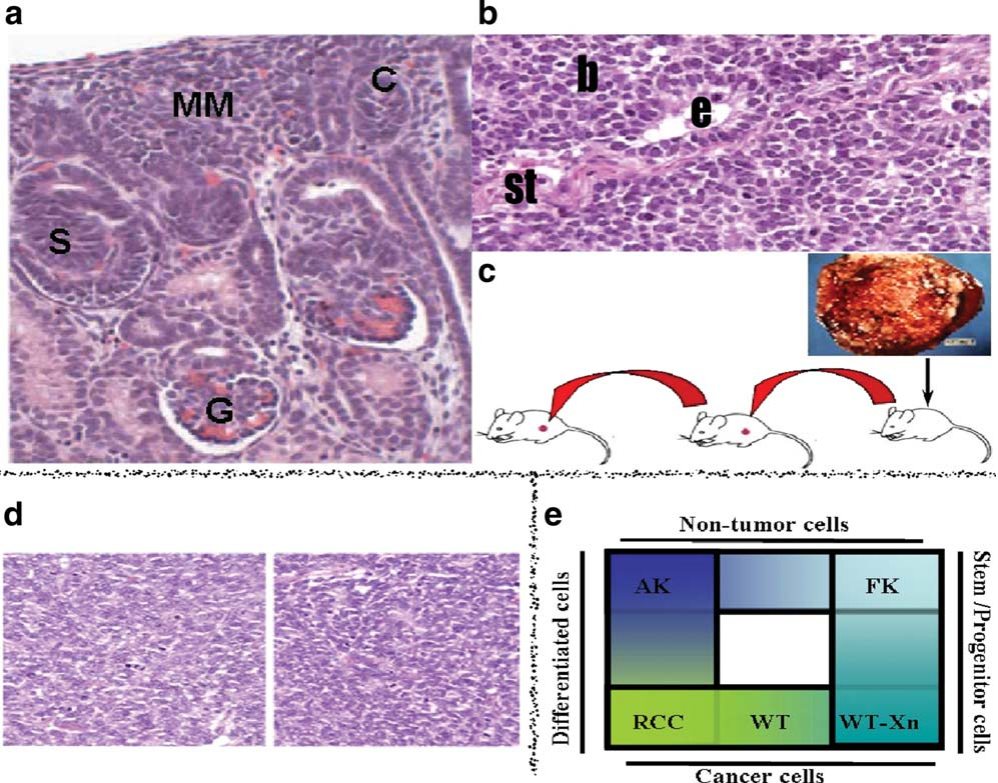
Strategy for the identification of human renal stem/progenitor markers. **(A):** Histological appearance of normal fetal kidney. **(B):** Histological appearance of primary WT. WT arises from multipotent renal embryonic precursors that undergo partial differentiation arrest, leading to a tri-phasic appearance of undifferentiated blastema (b) that resembles the MM, as well as differentiated tubular epithelial (e), and stromal (st) elements. **(C):** Establishment of WT-xenografts (Xn). Primary WT were implanted into SCID mice and then serially propagated, eventually leading to enrichment of stem/progenitor cells (blastema) at the expense of differentiated elements (seen in **[D]**). **(E):** Renal “stemness” markers are those elevated in microarrays of both stem-like WT-xenografts and human fetal kidneys, but not renal cell carcinoma or adult kidneys. Abbreviations: AK, adult kidney; C, C-shaped body; FK, fetal kidney; G, glomerulus; MM, metanephric mesenchyme; RCC, renal cell carcinoma; S, S-shaped body; WT, Wilms' tumor.

To circumvent this heterogeneous appearance, we serially propagated WT xenografts in mice. As a result, the progenitor blastema expanded at the expense of differentiated elements, creating stem-like tumors [18]. We hypothesized that overlapping overexpressed genes in WT-stem like tumors and developing human kidneys could serve as embryonic renal stem cell markers. Indeed, gene analysis uncovered a renal stemness signature set that included the nephron “progenitor” genes (*PAX2*, *EYA1*, *WT1*, *SIX1*, *SALL1*, and *CITED1*), *HOX* genes, *WNT* pathway and Polycomb group genes, and a limited number of surface markers (neural cell adhesion molecule 1 [*NCAM1*] [*CD56*], poly-sialated neural cell adhesion molecule 1 [*PSA-NCAM1*], *FZD7*, *FZD2*, *DLK1*, *ACVRIIB*, and *NTRK2*) [51] (Fig. 5).

These surface markers were exploited to define putative malignant renal stem/progenitor cells in primary WT cultures marked by NCAM1 but not CD133 [52], and they were also comprehensively characterized in the human fetal kidney to determine their relevance in pinpointing the human renal stem/progenitor cell pool and enabling its isolation via cell selection strategies [52]. Using this approach, we showed that immunoselection of cells from the human fetal kidney according to a combination of NCAM1 and EpCAM (CD227) demonstrated consistent overexpression of nephron progenitor genes, in particular *SIX2*/*OSR1*. In addition, high vimentin and low E-cadherin expression indicated that the cells have yet to undergo mesenchymal-epithelial transition to differentiated nephron epithelia [52]. Further characterization of isolated nephron progenitor cells is ongoing.

In our study, markers considered universal, such as CD133 and CD24, previously reported to identify renal progenitor cells in both embryonic and adult kidney [45,46], appeared mostly as markers for identification of differentiated renal epithelia among human fetal kidney cells. Therefore, the combination of these two markers is not likely to enrich a renal progenitor phenotype. Similarly, using an elegant transgenic mouse model, in which the endogenous promoters of *CD133* drive the expression of the reporter gene *lacZ*, and by immunohistochemical staining of mouse and human specimens, Shmelkov et al. [53] showed that CD133 expression in epithelial tissues is not restricted to stem or progenitor cells, but rather ubiquitously expressed on differentiated colonic epithelium in both mice and humans. To validate these results, the researchers examined expression of the *lacZ* reporter in the adult kidney, an organ previously reported to have large numbers of CD133^+^ cells [54], and found robust CD133 expression.

Thus, areas in the adult kidney previously reported to contain renal stem cells [45,46,55,56] are not necessarily remnants of the embryonic renal progenitors but rather contain differentiated cells with proliferating and even clonogenic capacities, as recently shown for differentiated pigmented ciliary epithelial cells, initially identified as retinal stem cells [57].

### Clonogenic Assays

An alternative to the initial step of stem cell isolation via sorting according to specific surface markers takes advantage of the fact that stem cells are highly clonogenic [58]. This approach, which starts from heterogeneous not enriched cell populations, requires assay systems that allow analysis of a single cell culture, as in the case of the neurosphere method for neural stem cells and the colony assay for hematopoietic progenitors [59]. Osafune et al. [60] set up an assay using *Wnt4* as an inductive signal, which could identify and characterize progenitor cells with multipotent differentiation potential from uninduced MM which could be used in the future for other cell sources. They found that only cells strongly expressing *Sall1*, isolated from *Sall1-GFP* mice, formed colonies that partially reconstituted a three-dimensional (3D) kidney structure, which contains glomeruli- and tubule-like components in an organ culture setting. This assay, however, has not yet been used for human kidneys.

In sum, renal progenitors isolated from the developing human kidney represent a promising source for allogeneic renal regeneration. Although methods to precisely define and isolate progenitors are currently being developed, selective culture conditions remain to be defined to enable retention of full developmental and regenerative potential upon expansion.

## ISOLATION OF RENAL PROGENITORS FROM DEVELOPED KIDNEYS

### Kidney Stem Cells in the Adult—Myth or Reality?

Many adult tissues are considered to harbor cells that self-renew and differentiate to form clones of stem, progenitor, and mature cells of the organ, fitting within the criteria of tissue-specific multipotential stem cells [61]. Some examples are the hematopoietic system, the skin, and the intestine [62–64].

In contrast to these rapidly cycling organs, the kidney has a low rate of cell turnover under steady-state conditions [58], and its regenerative capacity is limited. To date, there is no definite evidence for the existence in the adult kidney of a cell that fits within this definition. A kidney stem cell should be capable, at the clonal level, on the one hand to self-renew and differentiate into the nephron's cell types, and on the other hand contribute to renal repair by localizing and differentiating at sites of injury.

### Disconnecting Organogenesis and Regeneration

As discussed earlier, on completion of nephrogenesis, the MM/CM self-renewing renal progenitor population is entirely exhausted and therefore no progenitor population with nephrogenic potential similar to the CM exists in the adult. In this context, Hartman et al. [20] demonstrated in mice complete loss by postnatal day 3 of the CM. Interestingly, Humphreys et al. [65] not only showed lack of expression of the CM marker gene *Six2* in healthy adult mice kidneys, but also excluded the reactivation of this gene on the induction of ischemic kidney damage. These findings suggest that the CM population is not re-established postinjury by recapitulation of the developmental genetic pathways.

It appears therefore, that renal repair in the adult is established through replacement of necrotic tubular cells in surviving nephrons and not by the formation of new nephrons. A strong case is suggested for the replacement of tubular cells by proliferation of other differentiated tubular cells. This is, for example, the recognized mechanism in the pancreas [66]. Vogetseder et al. explored this mechanism during normal kidney homeostasis in the S3 segment of rats [67–69] and found that cycling and noncycling cells were both differentiated cells and that most tubular cells divide or enter the cell cycle in a period of 2 weeks, suggesting that a potential for proliferation exists in most, if not all cells of the S3 segment. It was shown that a large proportion of tubular cells are in the G1 phase, and that quiescent cells subjected to a mitotic stimulus re-enter the cell cycle, implying that tubular cells, many of which are in the G1 phase, are ready to respond to injury with a rapid proliferative response. In addition, it was shown in the ischemic kidney that the replacement of tubular cells involves dedifferentiation and proliferation of the surviving tubular cells [70,71]. Thus, both during normal kidney turnover and after damage, a valid option is replacement by mature tubular cells of their necrotic partners.

However, over the past few years, several groups have isolated from the adult kidney different cell populations harboring progenitor potential using various methodologies.

**Figure 6:**
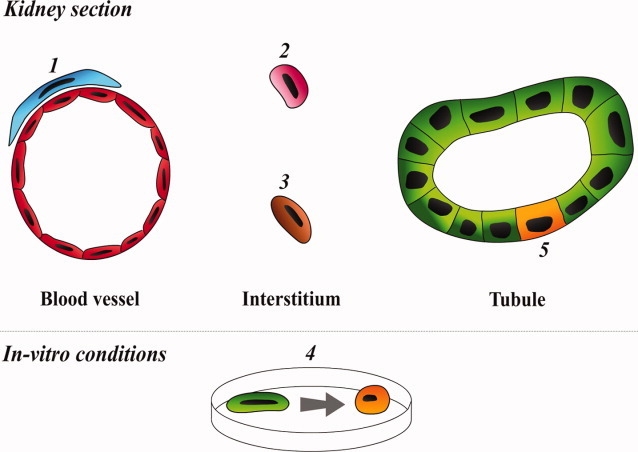
Possible explanations for the isolation of progenitor cells from developed kidneys. (1) and (2): Isolation of resident progenitors, for example, kidney MSCs (1, blue) or hemato-vascular progenitors (2, pink). (3): Isolation of a stromal progenitor cell (brown). (4): Isolation of a fully differentiated cell type (green) that acquires some progenitor properties on in vitro culturing (demonstrated by the transition in the culture dish into an orange cell type). (5): Isolation of tubular progenitors with a more restricted potential (orange).

The question that now arises is “what have we been isolating?” Various explanations, listed below and summarized in Figure 6, may account for this discrepancy.

#### Isolation of a Resident Progenitor Rather Than an Intrinsic Cell Type

Resident progenitors are defined as cells that do not originate from the MM and localize to the kidney's interstitial space such as bone marrow-derived cells. Resident progenitors are less likely to be relevant to kidney regeneration, as Humphreys et al. [65] demonstrated by lineage tracing that the cells responsible for tubular regeneration after ischemia are of tubular origin, thereby excluding an extrarenal source. An example for such a putative resident progenitor population is the renal MSCs.

MSCs, once hypothesized to be responsible for the homeostasis of adult mesenchymal tissues [72], are now considered a subpopulation of perivascular cells (or pericytes), residing in virtually every tissue [73–75]. MSCs probably contribute by recruitment from their perivascular niche to sites of injury, and by secretion of bioactive molecules, thereby establishing a regenerative microenvironment [74]. As blood vessels and pericytes vary among tissues, it is expected that MSCs from diverse tissue sources are also different [76]. Indeed, recent studies [77–79] confirmed this assumption.

It is therefore possible that each organ contains its own specific population of resident MSCs. For example, Da Silva Meirelles et al. [80] showed that long-term MSC cultures could be established from virtually every murine tissue including total kidney and kidney glomeruli [77]. Furthermore, MSC populations have been isolated from various fetal and adult human tissues [73], including the kidney [56].

#### Isolating an Intrinsic Stromal Progenitor Cell

The developing kidney contains at least two specific progenitor populations [10], that is, the Six2+ nephron progenitors and the Foxd1+ stromal progenitors, which represent mutually exclusive progenitor compartments. Remnants of the latter population in the adult kidney should be relatively easy to clone, passage, expand, and differentiate along mesoderm lineages in adhesive cultures, similarly to other stromal populations. Importantly, the Foxd1+ stromal population does not give rise to nephron epithelia [65] and lacks nephrogenic potential.

Recently, it has been demonstrated that interstitial cells, pericytes, residing within the adult kidney, are derivatives of the embryonic kidney's Foxd1+ stromal population, accounting for most of the myofibroblasts formed during renal fibrosis [81]. This finding demonstrates that interstitial cells do not contribute to tubular regeneration, and might even negatively affect the repair process.

#### Isolating a Fully Differentiated Cell Type with Some “Stem/Progenitor” Properties

Although shown to posses progenitor properties, it is possible that some of the populations isolated were in fact differentiated cells. Several facts support this notion.

First, adult differentiated epithelial cell types have been shown to possess clonogenic and self-renewing capabilities leading to their possible misinterpretation as stem cells/progenitors [57].

Second, ex vivo growth conditions of cells may result in a nonspecific phenotypic switch of differentiated epithelial cells during epithelial-mesenchymal transition (EMT). Although these cells may show enhanced proliferation and migration and appear in a progenitor state, their nature is mostly fibroblast/mesenchymal-like, lacking functional relevance [82].

A third reason for this possible misinterpretation is the use of surface markers or functional parameters for isolation that overlap with those of differentiated cell types or that actually mark only differentiated cells. Examples of such cell markers include “universal” stem cell markers such as CD133, CD24, Sca-1, and c-Kit, which have all been shown to be heavily expressed in differentiated epithelia, including renal epithelia [52–54,83–85]. Examples of overlapping functional parameters are those used for HSC isolation (label retention and dye efflux capacity) that do not discriminate between progenitors and differentiated cells in other organs [58].

Fourth, the lack of appropriate controls for an alleged progenitor cell fraction can also lead to confusion. Analysis of expression levels of pluripotency or renal developmental markers, clonogenicity, multipotentiality, and in vitro and in vivo differentiation potential in a specific cell type are irrelevant if not compared with a cell not expressing the alleged progenitor phenotype, demonstrating advantageous properties or function.

Renal potential should be inherent to the biology of a renal stem cell. Clearly, lack of a robust in vitro assay to analyze nephrogenic potential at the single cell level (as achieved by limiting dilution), as opposed to the often performed mesenchymal tri-lineage (adipocytes, chondrocytes, and osteoblasts) differentiation assay relevant for MSCs (but not to renal progenitors), limits the exclusion of differentiated cells and the inclusion of a bona fide renal stem cell. For such an assay, developmental cues driving nephrogenesis, as stated earlier, are likely to be relevant.

In addition, although in vivo renal potential can be studied in models of renal damage (acute and chronic) or preferably in models of metanephric development in which the microenvironment can support, at least in part, differentiation, one must exclude cell fusion to establish unequivocal renal potential.

#### The Kidney Harbors a Progenitor Population That May Function Through Genetic and Differentiation Pathways Other Than the Ones Active During Embryonic Nephrogenesis

A population with a more restricted potential than the CM (e.g., a progenitor cell type for proximal tubular cells) may exist. This option might be supported by the finding that many developmental genes are upregulated after kidney damage [49,51,86,87], indicating the possibility that partial recapitulation of development might occur. However, such populations might be too small to elicit measurable regeneration and assist in renal repair, leading to two scenarios for clinical translation.

The first is in vitro expansion with risks of cell differentiation or mal-differentiation including acquisition of mutations and possible malignant transformation.

Alternatively, inducing proliferation of progenitor cells within their native niche in the kidney via delivery of soluble factors/drugs or other cell types is an option carrying the inherent advantage of sparing the patient a renal biopsy. An example for such cells is MSCs, as one of the presumed mechanisms for their paracrine effect on the kidney is recruitment of local stem cells [47].

Summarizing the above arguments, a list of reports identifying cells with progenitor potential in the adult kidney are presented (Table 1 and Supporting Information).

**Table 1:** Methods used to isolate progenitors from the adult kidney (see Supporting Information for details)

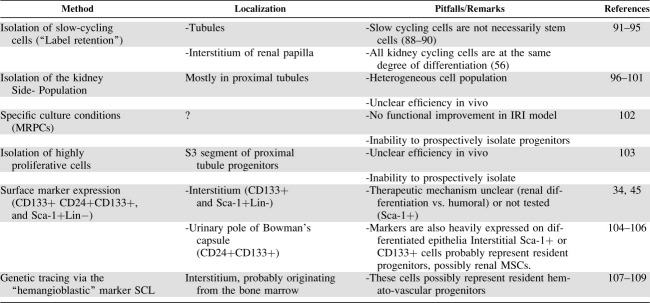

Abbreviations: IRI, ischemia-reperfusion injury; MRPCs, multipotent renal progenitor cells; MSCs, mesenchymal stromal cells; SCL, stem cell leukemia.

## REPROGRAMMING RENAL PROGENITORS

As discussed previously, the current body of evidence strongly suggests that no residual progenitor population of the CM resides in the adult kidney, limiting its regenerative capacities [10]. Thus, instead of investing efforts in the isolation of such cells from the adult kidney, one can propose to artificially create CM and nephron progenitors from mature cells, maintaining the advantage of an autologous cell source. Because few attempts, if any, have been made to use this strategy for kidney regeneration, we will focus on general principles that, in the future, could be used to generate reprogrammed cells for kidney repair.

The consensus held today is that under physiologic conditions, the fully differentiated state of a cell in the adult is permanent, and therefore reprogramming must be forced on cells [110]. Furthermore, early studies showing cellular plasticity in adult stem cells have been challenged [111]. Nonetheless, by applying experimental tools, today, biology is able to force cells to switch from one type to another. For instance, complete dedifferentiation into pluripotency [112–114] or transdifferentiation between different types of cells, whether differentiated or stem cells [115–118] has been reported. Thus, any cell can probably be reprogrammed into any another type of cell, given expression of the right transcriptional network.

Similar to any reprogramming protocol, when trying to achieve renal reprogramming, a few important questions must be answered.

### Which Transcription Factors Are Relevant?

It has been demonstrated [112,115,116] that a limited number of transcription factors introduced into cells is capable of activating the full transcriptional machinery necessary for converting cell fate. Many examples of reprogramming relied on re-expression of developmental genes [119–121].

In the kidney, the genes to be activated depend on the identity of the cells we are trying to create. Will we be trying to achieve mature, functional cells (maybe in situ [115]) or rather a progenitor cell that following differentiation will achieve neo-nephrogenesis in the adult? The latter option seems preferable because unlike other organs (e.g., pancreatic β-cells), the kidney relies on the orchestrated function of various cell types within a specific 3D structure.

Therefore, kidney regeneration will probably require a multipotent cell population capable of replenishing the full spectrum of cells. If this is the case, what is the specific embryonic population we are looking for? The answer to this question is not trivial, as the kidney sequentially develops from posterior IM, through MM and CM and up to the fully developed nephron, each precursor cell type possessing its own differentiation potential and a typical gene expression pattern. In addition, different diseases might require different cell types (podocytes, tubular cells, etc.). A reasonable option is to reprogram cells into a MM/CM-like state, as this is the direct precursor tissue of the nephron. Clearly, if this is the population we are searching for, continuing efforts to decipher the phenotypical identity of the human renal progenitor population within the MM/CM are crucial.

Which genes are relevant? The molecular mechanisms governing kidney development, intensively investigated during the last 20 years, enable us to wisely choose the appropriate reprogramming factors. Although numerous genes regulate kidney development and cell lineages [10], those exhibiting a clear knockout phenotype are probably best suited for reprogramming (including *Osr1*, *Lim1*, *Pax2/Pax8*, *Wt1*, *Foxd1*, *Hox11*, *Six1/Six2*, *Sall1*, and *Eya1* [14,91,122–129]). This list can be narrowed, as done recently by Zhou et al. [115], by testing the candidate factors in different combinations until the right one is found to induce optimal reprogramming.

Additional Augmentation Techniques for Reprogramming Are Briefly Covered in the Supporting Information.

### Which Cell to Reprogram?

The second aspect of reprogramming is the choice of cells to be reprogrammed.

Undoubtedly, some cell types are better candidates than others, with the main criterion being developmental proximity between the cell types [110], as this reflects the differences in the epigenome that will have to be encountered in order to activate the correct set of genes. In this case, good candidates might be adult kidney epithelia or, if identified, uni-potential progenitor cell populations in the adult kidney. Nonetheless, more developmentally distant cells cannot be excluded.

### How to Prove That the Conversion Was Successful?

Finally, the importance of demonstrating a full phenotypic and functional change into the desired cell type will be discussed.

Unlike nondesirable EMT resulting in a nonspecific fibroblastic phenotype, the induced cell should undergo reverse nephrogenesis to a mesoderm phenotype. This new cell type should upregulate the renal progenitor genes, culminating in a stable progenitor-state amenable to in vitro and in vivo inductive signals, preferably at the clonal level, to preserve nephrogenic potential. It is prudent to exclude cell fusion and a hybrid phenotype, that is, upregulation of only a few genes due to overexpression of potent transcription factors.

## CONCLUSION

Reports of kidney stem cell populations in mouse and human kidneys are met with enthusiasm because of their potential for cell-based therapies to treat millions of people with renal failure worldwide. Nevertheless, to date, the presence of a true adult kidney stem cell remains elusive.

This does not eliminate the possibility of using sorted, clonogenic, or in vitro expanded populations of adult kidney cells as cell-based therapies. In addition, a functional benefit may arise from various cell types lacking nephrogenic potential.

Efforts should be directed toward replenishment of nephrons through isolation of progenitor cells from fetal kidneys, reprogramming them from adult cells or using differentiated ESCs/iPS cells.

## Disclosure of Potential Conflicts of Interest

The authors indicate no potential conflicts of interest.
