# Supplementary figures and images for: Concise Review: Kidney Stem/Progenitor Cells: Differentiate, Sort Out, or Reprogram?
Source: Stem Cells. 2010 Jul 22;28(9):1649–60. doi: 10.1002/stem.486 (PMC2996087; doi:10.1002/stem.486)

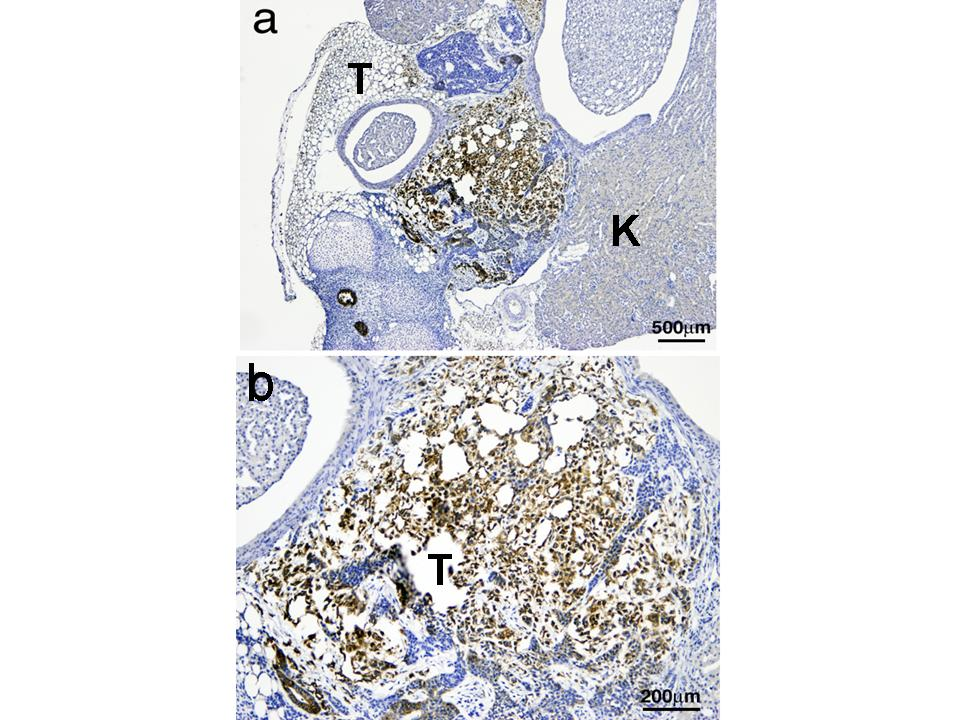

Supplement: Supplementary file 1 [file stem0028-1649-SD1.tif]

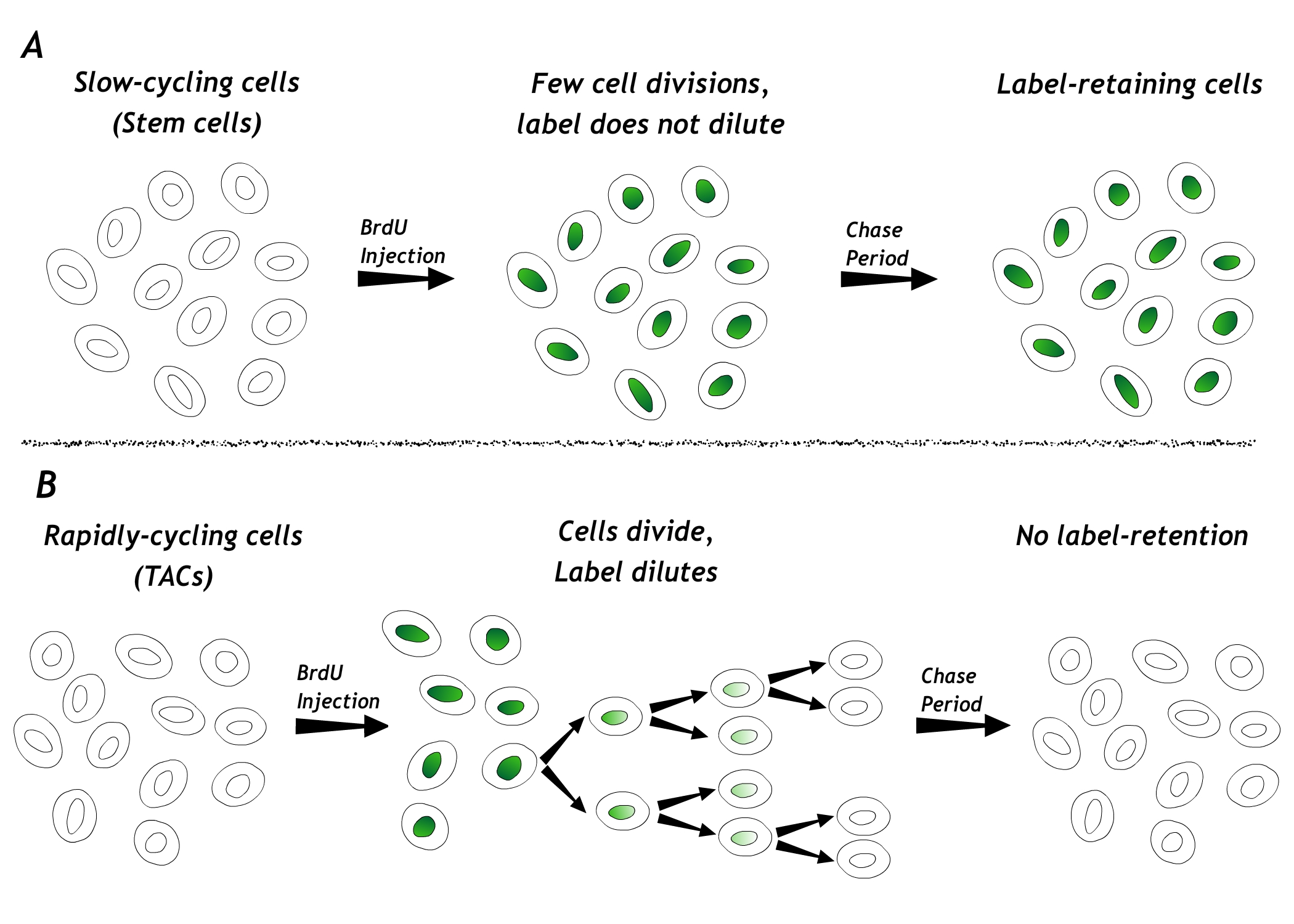

Supplement: Supplementary file 2 [file stem0028-1649-SD2.tif]

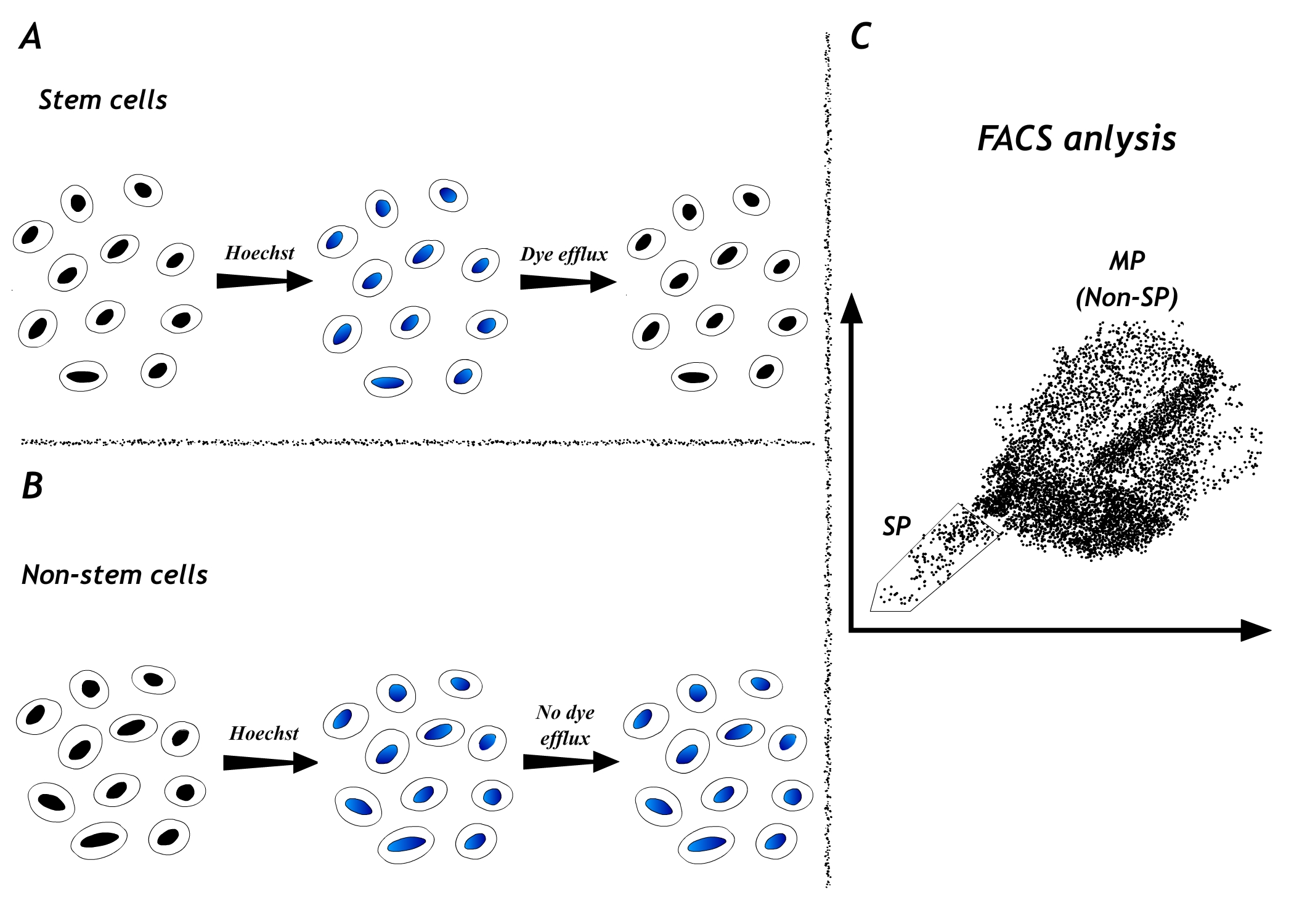

Supplement: Supplementary file 3 [file stem0028-1649-SD3.tif]

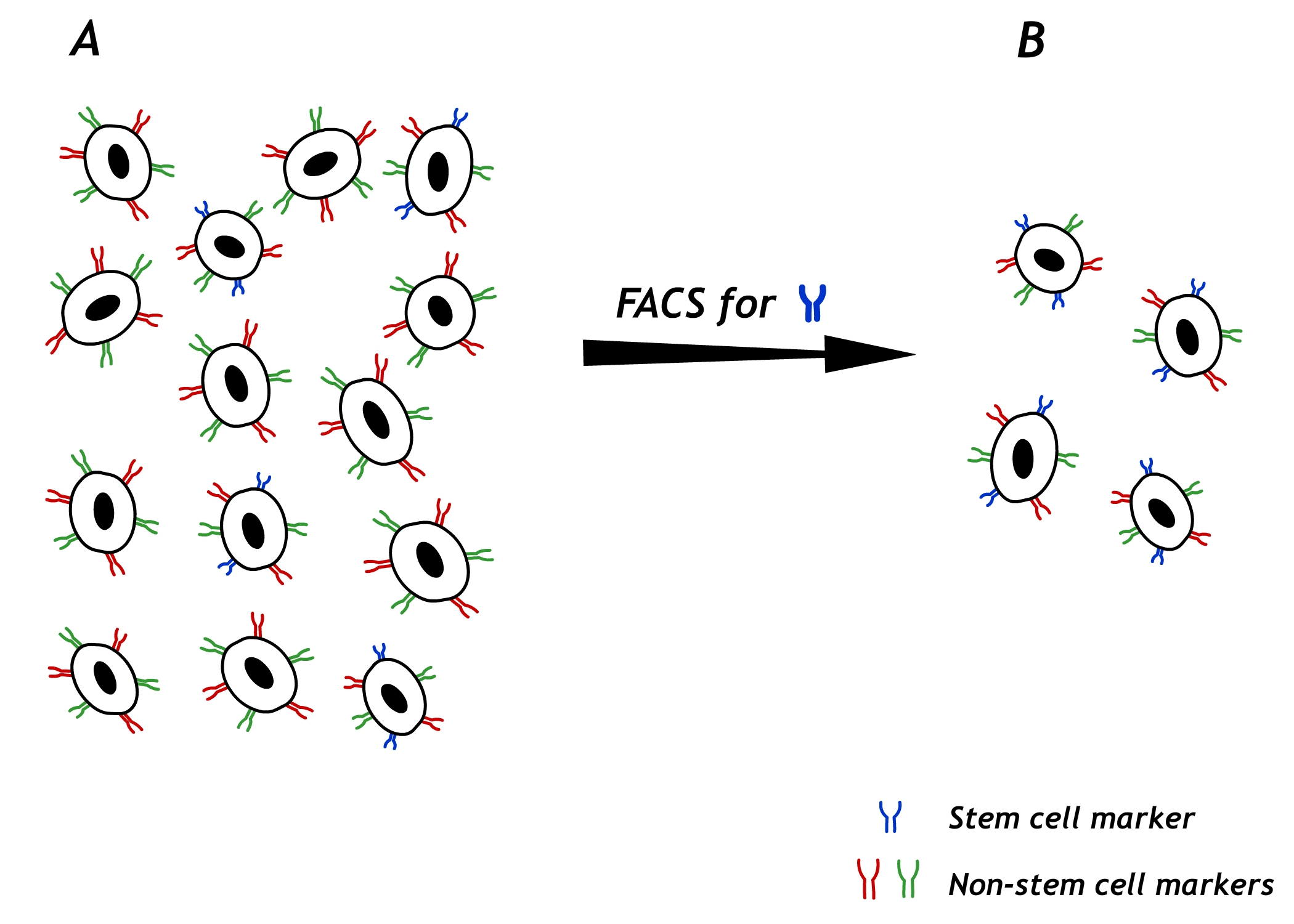

Supplement: Supplementary file 4 [file stem0028-1649-SD4.tif]

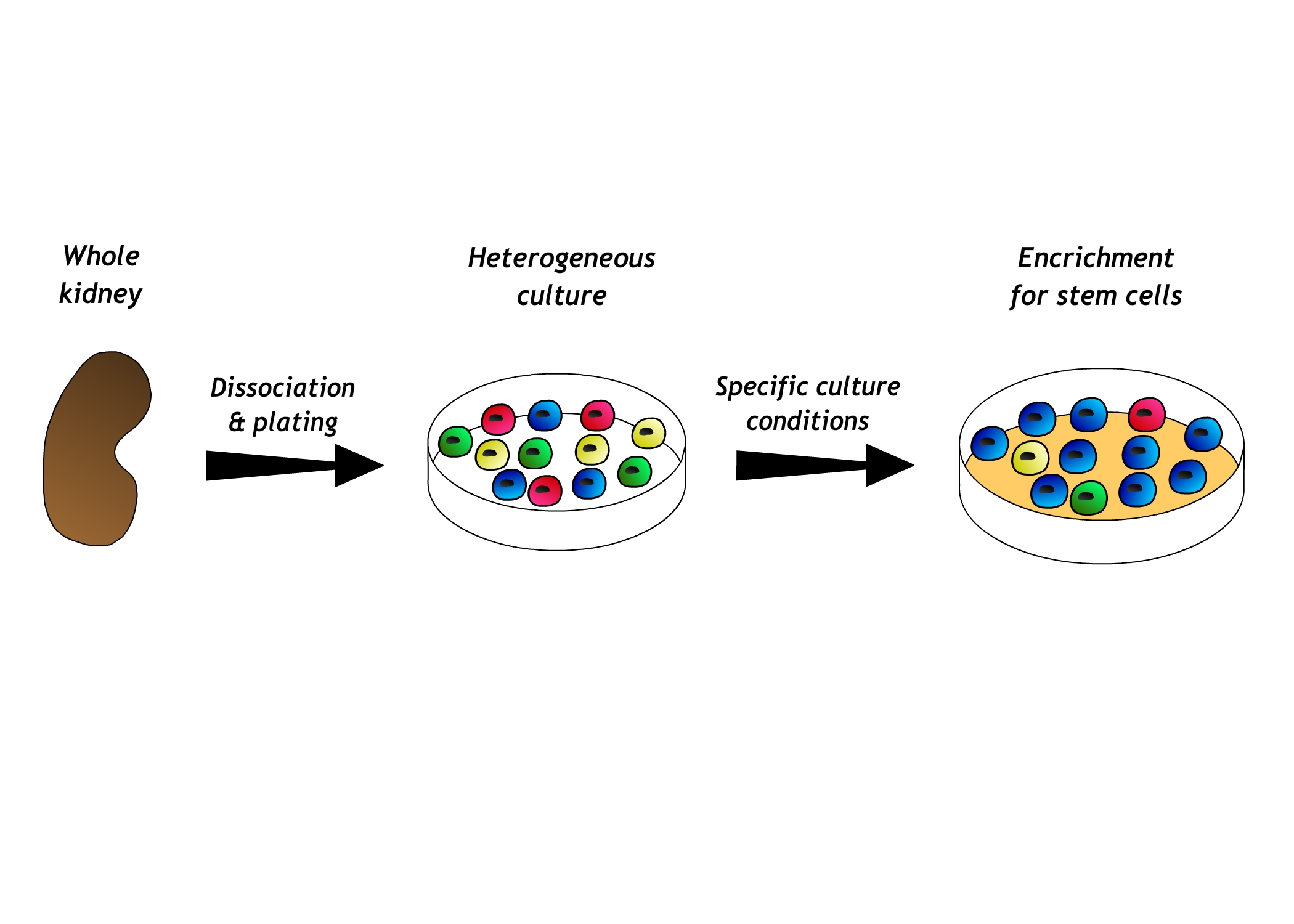

Supplement: Supplementary file 5 [file stem0028-1649-SD5.tif]
