# Supplementary material for: Concise Review: Kidney Stem/Progenitor Cells: Differentiate, Sort Out, or Reprogram?
Source: Stem Cells. 2010 Jul 22;28(9):1649–60. doi: 10.1002/stem.486 (PMC2996087; doi:10.1002/stem.486)
Supplement: Supplementary file 6 [file stem0028-1649-SD6.doc]

**Supplemental data:**

***Methods for isolation of progenitor cells from developed kidneys***

***1. Isolation of slow-cycling cells:***

One of the earliest approaches to identification of kidney stem cells relied on their allegedly slow-cycling nature, a property which is thought to enable them, as the cells responsible for the homeostasis of the entire tissue, to minimize mutation rate (58).

Therefore, a common strategy to identify stem cells consisted of a brief exposure of cells to BrdU, a dye that incorporates into the DNA of dividing cells, and examination the cells after varying periods of time (chase periods) for the presence of the dye. As every cell division dilutes the dye, slow-cycling cells retain BrdU, whereas in rapidly-dividing cells, the dye quickly dilutes and becomes undetectable (see **supplemental Figure 2**). This method was used to identify putative stem cells in different tissues (109-114).

Maeshima et al. were the first to identify label-retaining cells (LRCs) in the renal tubuli of normal rat kidneys (115), which they designated LRTC (label retaining tubular cells) proving that they are the major source for proliferating cells during recovery from ischemic damage. In addition, LRTC participate in UUO (unilateral ureteric obstruction)-induced renal fibrosis, by proliferating, migrating to the renal interstitium and acquiring a fibroblast-like phenotype (116). Utilizing FACS to isolate viable BrdU-positive cells (relying on the fact that the fluorescence intensity of Hoechst 33342 is reduced by the incorporation of BrdU into the DNA), the same group showed that these BrdU positive/Hoechst low cells posses phenotypic plasticity as well as the ability to form tubule-like structures and integrate into embryonic rat metanephroi (117).

In contrast, Oliver et al. (118), using longer chase periods, found that the kidney LRCs reside in the *interstitium* of the renal papilla, and that after an ischemic insult, these cells proliferate and disappear from the papilla, with very little apoptosis taking place, suggesting that they participate in the repair process. Very recently, the same group demonstrated that LRCs of the papilla proliferated only in its upper part, where they associated with ‘chains’ of cycling cells and that papillary cells, LRCs included, migrate during normal homeostasis towards different parts of the kidney (119). Kidney injury enhanced both migration and proliferation of the cells, suggesting a role for these papillary LRCs both during normal homeostasis and after injury.

There are several limitations to the label retention approach. First, this is a destructive procedure that does not allow for prospective isolation of the LRCs. Second, because BrdU only incorporates into dividing cells, it is unlikely that this method will enable identification of the entire stem cell population. Additionally, the assumption that slow-cycling cells are stem cells has been recently challenged (120-122). Furthermore, Vogetseder et al. (67) showed that all cycling cells in the rat kidney show similar degree of differentiation compared to non-cycling cells and that most LRCs are differentiated cells, suggesting that label retention is probably not a criterion for identification of stem cells in the kidney.

***2. Isolation of the kidney side-population:***

A second approach for finding kidney stem cells was to investigate the kidney's side population (SP). This approach derives from the method described by Goodel et al. (123) for isolation of murine HSCs (hematopoietic stem cells), based on their ability to efficiently efflux a fluorescent dye. The cells are subjected to Hoechst 33342 dye staining and then sorted by FACS. Cells able to actively efflux the dye appear as a distinct cell population on the side of the Hoechst blue vs. Hoechst red plot (**supplemental Figure 3**).

Cells with the SP phenotype have been isolated from a wide range of tissues (124-130). Several studies exploring the kidney SP reported different results regarding its size, differentiation potential and origin (intra- vs. extra-renal) (131-134).

Asakura et al. (131) found a kidney SP in the mouse, comprising 5% of total kidney cells, most of them CD45+ and therefore probably BM (bone marrow)-derived. In contrast, Iwatani et al.(132), described a population comprising 0.03 - 0.1% of total cells in the rat kidney, with little renal potential and only a small contribution of BM to the population, as only 10% of the kidney SP of rats that underwent a BM transplant from a GFP+ donor were GFP+. Hishikawa et al. (133) estimated the kidney SP to be about 5.1% of total kidney cells in mice. He further demonstrated functional improvement in cisplatin-induced AKI (acute kidney injury) but not in models of chronic damage, and described up-regulation of renoprotective factors. In addition, MyoR was described as a potential marker for this population.

In an attempt to clarify these contradicting results, Challen et al. (134), analyzed mouse fetal and adult kidneys and found that the kidney SP represents about 0.14% of the total cells, is localized mostly in the proximal tubules and exhibits enhanced proliferation compared to the rest of the kidney cells. In addition, low expression of hematopoietic markers was noted. This fact, together with the relatively similar profile of gene expression between fetal and adult kidney SP implied it is a resident rather than BM-derived population (although this conclusion needs to be validated by lineage tracing studies). When injected into mouse embryonic metanephroi, SP cells showed a much higher engraftment potential into MM (metanephric mesenchyme) and UB (ureteric bud)-derived structures than the main population (MP). *In-vivo*, kidney SP cells led to functional improvement in adriamycin-induced AKI (though not significant advantage over MP cells was demonstrated), but without proof of engraftment into the kidney, which led the authors to suggest a primarily *humoral* mechanism of action. In this context, Imai et al. (135) reported that the basal expression of several renoprotective factors, such as BMP7, VEGF, and HGF is higher in SP than in non-SP cells. Recently, Inowa et al. (136) proved the existence of a *human* SP, representing 1.3% of total kidney cells.

In conclusion, the kidney SP is a heterogeneous population, with unclear efficiency *in vivo*, that acts mostly through humoral mechanisms. The dynamic nature of the parameter being measured (dye efflux) will probably lead to significant variations in the cell populations being isolated, making the use of this method *alone* impractical for stem cell isolation.

***3. Isolation via surface marker expression:***

The first work attempting to isolate stem cells from adult kidneys based on surface marker expression, by Bussolati et al. (137), demonstrated that CD133+ cells negative for hematopoietic markers from adult human kidneys represent about 1% of total kidney cells and are located in the renal interstitium, potentially representing stromal cells. This population was found to express Pax2 and was capable of epithelial and endothelial differentiation both *in vitro* and *in vivo*. When injected into SCID mice with glycerol-induced AKI, the cells integrated into tubuli.

Sagrinati et al. (46) isolated a subset of parietal epithelial cells (PEC), localized in the urinary pole of Bowman’s capsule, based on the co-expression of CD24 and CD133, which they named APEMPs (adult parietal epithelial multipotent progenitors). These cells, representing 0.5-4% of cortical renal cells, demonstrated self-renewal and enhanced clonogenicity and lacked the expression of renal-specific markers. In addition, they demonstrated multipotentiality both *in vitro* and *in vivo*, including towards the renal lineage (as demonstrated, at least in part, by the up-regulation of markers of different portions of the nephron under specific culture conditions). When injected into mice with glycerol-induced AKI, the cells localized to tubules and reduced morphologic and functional kidney damage compared to CD133-CD24- cells. Of note, murine CD24a, suggested as a renal progenitor marker (48), and human CD24 are not orthologues.

Ronconi et al. (55) further characterized the APEMPs and showed that this is a hierarchical and heterogeneous population of cell types, arranged in a specific manner inside Bowman’s capsule. Cells exclusively localized to the urinary pole, expressing CD24 and CD133 but not the podocyte marker PDX (CD133+CD24+PDX- cells) as opposed to more differentiated and less clonogenic cell types (CD133+CD24+PDX+ and CD133-CD24-PDX- cells) can regenerate both tubular cells and podocytes. CD133+CD24+PDX- cells proved useful in adriamycin-induced renal damage in SCID mice, reducing proteinuria and urinary albumin/creatinine ratio. The study did not address the question of whether the mechanism of improvement was integration and trans-differentiation or rather humoral. Taking into account that podocyte depletion is a determining factor in many kidney diseases (138-140), Ronconi et al (55) provide insights into a possible source for podocyte regeneration. Supporting this finding, Appel et al. (141) showed that at least some of the cells responsible for podocyte regeneration during normal homeostasis are derived from the PEC (parietal epithelial cell) population of the glomeruli. They described a transitional cell population with morphologic and immunohistochemical features of both PECs and podocytes, situated at the glomerular vascular stalk. When rats were injected with BrdU, which labeled only dividing PECs and not podocytes, a 2-3 fold increase in BrdU-labeled podocytes was noticed, suggesting that the PECs are the cells responsible for the replacement of podocytes. Finally, using a triple-transgenic mouse model, in which PECs were specifically and irreversibly labeled, it was shown that the number of labeled glomerular cells increases with time and that these cells co-express podocyte markers.

This method for stem cell isolation is illustrated in **supplemental Figure 4**.

***4. Other methods:***

Gupta et al. (142) , again relying on previous studies on BM stem cells, used culture conditions similar to those used to isolate MAPC (multipotent adult progenitor cells) from the BM, muscle and brain (143) (**supplemental Figure 5**). After culturing dissociated rat kidney for 4-6 weeks, spindle- shaped fibroblast-like cells, demonstrating self renewal and expressing Oct4 and Pax2 appeared in the culture. Interestingly, these cells were NCAM1- and CD133-. In response to a nephrogenic cocktail (FGF2, TGFβ and LIF), the cells formed aggregates, up-regulated epithelial markers and down-regulated Oct4, while maintaining Pax2 expression. *In vivo*, the cells integrated into the kidney, proliferated, expressed markers characteristic of proximal and distal tubules and formed multiple tubular-like structures. However, no functional improvement was observed in an IRI (ischemia-reperfusion injury) model.

Kitamura et al. (144) used a different approach and by culturing cells from different segments of the rat kidney obtained by micro-dissection, searched for highly proliferative cells. By repetitive plating of cells after limiting dilution, they isolated and characterized a cell line from the S3 segment of the proximal tubule, designated rKS56. This cell line expressed mature tubular markers such as AQP1, AQP2, and also vimentin, Sca1, Pax2 and GDNF, indicating a rather undefined, intermediate phenotype between a progenitor and a fully differentiated cell. *In vivo*, these cells created tubule-like structures and integrated into tubules in an ischemia model of AKI, but without substantial functional benefit.

***5. Kidney stromal cells:***

We have been the first to show in mice the presence of a multipotential clonogenic non-tubular cell fraction, isolated by immunoselection and exclusive FACS gating, and expressing Sca1 (145). Several characteristics suggest a progenitor MSC (multipotent mesenchymal stromal cells)-like population rather than an intrinsic renal stem cell: Expression of surface antigens (Sca1+CD29+CD45-Lin-), failure of gene profiling to show over-expression of renal progenitor genes (in contrast to their high expression in 'true' renal progenitor populations (49)), differentiation of a clonogenic population to fat and bone, and the ability to mediate an immunomodulatory effect.

More recently, Huang et al. (146) isolated a similar population of murine kidney-derived cells with morphologic features, growth properties and an immunophenotype characteristic of MSCs (Sca1+CD29+CD45-Lin- but also CD44+, most probably due to different culture conditions). They too, showed immunomodulation with these cells of dendritic- and T-cell responses.

Bruno et al. (56) described a similar resident MSC population in human adult kidney. They cultured decapsulated glomeruli and noticed that after three weeks, a monomorphic spindle-shaped population of cells took over the culture. This population was CD146+ and CD133-, exhibited a surface marker expression pattern characteristic of BM-MSCs and expressed vimentin but not epithelial markers. The cells expressed Nanog and Musashi but not Oct4 and displayed self-renewal and clonogenicity. *In vitro*, they differentiated into adipocytes, chondrocytes, osteoblasts, endothelium, mesangial-like cells and epithelial cells expressing specific podocyte markers, while losing the 'stemness'-related and mesenchymal markers during differentiation. Under the same conditions, BM-MSCs failed to differentiate into podocyte-like cells. In contrast to BM-MSCs, these cells expressed CD24 and Pax2, and when cells from the kidney of a male donor were transplanted into a female recipient, no cells with female nuclei were detected upon isolation of the population from the recipient, suggesting that this is a resident population (that may have developed *in-situ* or migrated from the BM early during kidney development).

In conclusion, it seems that the kidney harbors a population of resident MSCs that possibly contributes to kidney homeostasis and/or regeneration. Further studies are needed in order to test whether or not these cells are of clinical benefit and whether they operate in the kidney like BM-MSCs injury via humoral mechanisms.

***6. Kidney hemato-vascular progenitors:***

A second type of resident progenitors in the adult kidney, distinct from resident MSCs are the vascular progenitors. These cells contrast with circulating blood cells that have been shown to carry vasculogenic and not tubulogenic potential in diseased kidneys (20).

Early in mammalian development, the SCL/TAL1 (stem cell leukemia) gene and its distinct 3’ enhancer (SCL 3’En) specify bipotential progenitor cells, termed hemangioblasts, that give rise to both blood and endothelium (147). Using a reporter gene linked to SCL 3' En, enabling the detection of its activity, we previously found a minor population of SCL+ cells in the adult kidney of SCL 3' En transgenic mice (25). Lineage tracing showed that this population comprises three phenotypically different sub-populations: CD45+CD31- hematopoietic cells, CD45-CD31+ endothelial cells and, surprisingly, also a relatively large *CD45-CD31- interstitial* cell population, potentially signifying an angioblastic/hemangioblastic progenitor population (21). No such population has been shown to exist in the blood.

With the creation of BM chimeras (SCL 3' En BM into wild type host) all three populations could be traced back to the bone marrow. This does not rule out the possibility of these cells migrating to the kidney from corresponding areas during embryonic development. The near absence of such a SCL+CD31-CD45- cell fraction from the liver of chimeric mice demonstrated its specific predilection to the kidney.

When IRI was induced in transgenic mice, early (48h post-ischemia) elevation of SCL+ cells, mostly of the hematopoietic fraction, was detected. In addition, a week after the ischemic injury, a modest but significant increase in the SCL+ non-hematopoietic fraction was noticed, raising the possibility that this population is induced to proliferate post-ischemia.

Interestingly, Garcia-Ortega et al. (148) showed very recently that when fetal liver (FL) hematopoietic progenitors derived from SCL 3' En transgenic mice create chimeras in wild type hosts they are capable of forming a whole network of vascular-like patches in the kidney. Again, the kidney patches contained all three sub-populations described previously, including robust engraftment of the interstitial SCL+CD31-CD45- fraction. Further characterization of this sub-population demonstrated high CD34+ expression and lack of alpha-actin, consistent with a hemato-vascular progenitor.

Improved characterization of these cells will require their isolation from transgenic kidneys. However, the existence of such a population is consistent with the isolation of HSCs from different extra-medullary sites (149-151), including the kidney (152), and in accordance with the emerging concept that the BM is not the only niche for HSPCs (hematopoietic stem and progenitor cells), which constitutively survey extra-medullar non-lymphoid tissues, possibly contributing to the continuous restoration of specialized hematopoietic cells that reside in peripheral tissues (152).

Interestingly, Stroo et al. (153) demonstrated very recently that systemically injected HSC preferentially migrate to ischemic kidneys rather than to healthy ones, and that this process is independent of SDF1/CXCR4. Although this pathway was previously thought to be necessary for migration to ischemic areas (154), this finding opens the possibility that the kidney indeed possesses a niche for HSCs (155).

It should be noted that many studies in different organisms suggest that during embryonic development, the kidney is able of providing the microenvironment necessary to sustain hematopoiesis (reviewed in 155). Furthermore, definitive HSCs, hemangioblasts and very early embryonic renal progenitors share a common developmental origin – the aorto-gonado-mesonephros (AGM) region (156), further linking blood and kidney.

***Reprogramming renal progenitors:***

*Augmenting techniques for reprogramming*

Although most studies attempting to reprogram cells into different lineages utilized for this purpose over-expression of transcription factors, mostly via viral vectors (90, 93), it is important to mention the growing interest in other factors that can promote reprogramming.

Micro-RNA (miRNA), for example, may play an equally important role in reprogramming as transcription factors (157). Judson et al. (158) have shown that introduction of miRNAs specific to ESCs (embryonic stem cells) enhances mouse iPS (induced pluripotent stem) cell production, and Lin et al. (159) demonstrated that miR-302 reprograms human skin cancer cells into a pluripotent ESC-like state. Interestingly, Goodall and colleagues (160) demonstrated that EMT (epithelial-mesenchymal transition), a process that is relevant to kidney disease and also to potential reprogramming, is regulated by the miR-200 family and miR-205. Additionally, the miR-30 family regulates Xenopus pronephros development and targets the transcription factor Xlim1/Lhx1 (161).

Although further screening and characterization of miRNA expression patterns are needed, this appears as a promising new strategy for reprogramming.

Additional molecules that can assist in augmentation of reprogramming factors are chromatin-modifying agents, e.g. HDAC (histone deacetylase)-inhibitors (e.g. valproic acid). These agents have been shown to increase reprogramming efficiency and can even replace one or more of the transcription factors used for reprogramming (162,163). The reason is probably that chromatin remodeling is a rate limiting step in genetic reprogramming (162).

This ’chemical approach’ to reprogramming offers the advantages of reversible effects, the possibility to fine-tune the reprogramming effects via varying concentrations, and a high degree of temporal control. The major disadvantage, however, is a rather non-specific effect, targeting different families of proteins (164).

Combined with genetic approaches, these techniques should facilitate more effective and therefore clinically applicable reprogramming, and enable reprogramming events not otherwise possible. Importantly, epigenetic modifications responsible for transcriptional and lineage control, including within the renal lineage (17, 165), are reversible and can be therefore manipulated to enhance developmental competence of cells.

**Supplemental figures legends:**

***Supplemental Figure 1:*** Pluripotent human embryonic stem cell transplantation into ischemically injured mouse kidneys induces the formation of intra-renal teratoma (T) arising from the kidney's pelvis (K), shown at x10 (a) and x40 (b) magnification. Teratoma formation is one of the major risks of using pluripotent cells as starting material for kidney regeneration, underscoring the importance of inducing proper differentiation of the cells prior to their injection.

***Supplemental Figure 2:***

**Label-retention method:** Animals are injected with bromodeoxyuridine (BrdU) that incorporates into the DNA of dividing cells (represented by the green nuclei). In rapidly-dividing cells (**A**), the dye is diluted and after a period of time (chase period) becomes undetectable. In contrast, in slow-cycling cells (**B**) the dye remains detectable and therefore these cells are called label-retaining cells (LRCs). Whereas stem cells are assumed to be slow-cycling, transit amplifying cells (TACs) are rapidly-dividing cells and therefore LRCs are assumed to represent only stem cells.

***Supplemental Figure 3:***

**Isolation of the side population:**

Stem cells have been attributed the ability to actively efflux the fluorescent dye Hoechst 33342 *via* membrane transport pumps. Therefore, when exposed to it, only stem cells (A) efflux the dye, whereas other cells (B) retain it (demonstrated by the blue nuclei). Upon analysis via flow cytometry (C) the Hoechst-low cells appear as a distinct population of cells on the side of the profile, and are therefore called "side population" (SP).

***Supplemental Figure 4:***

**Isolation via surface markers:** The most accurate method to isolate stem cells is to use a marker known to specify them (blue surface marker), in order to sort them out using FACS (fluorescence-activated cell sorting) analysis. Currently, the lack of such specific antigens limits this option.

***Supplemental Figure 5:***

**Isolation via specific culture conditions:**

Dissociated kidney cells are grown under specific culture conditions, derived from those used to isolate MAPC (multipotent adult progenitor cells) from the bone-marrow and additional tissues. After several weeks the culture, which was initially heterogeneous, becomes enriched for cells with stem cell properties (blue cells).
